# A comparison of sex-specific immune signatures in Gulf War illness and chronic fatigue syndrome

**DOI:** 10.1186/1471-2172-14-29

**Published:** 2013-06-25

**Authors:** Anne Liese Smylie, Gordon Broderick, Henrique Fernandes, Shirin Razdan, Zachary Barnes, Fanny Collado, Connie Sol, Mary Ann Fletcher, Nancy Klimas

**Affiliations:** 1 Department of Medicine, University of Alberta, Edmonton, AB, Canada; 2 Department of Medicine, University of Miami, Miami, FL, USA; 3 Department of Clinical Immunology, Miami Veterans Affairs Medical Center, Miami, FL, USA; 4 Institute for Neuro-immune Medicine, Nova South eastern University, Suite 3440 University Park Plaza, 3424 South University Drive, Fort Lauderdale, 33328, FL, USA

**Keywords:** Cytokines, Chronic fatigue, Gulf war illness, Exercise challenge, Immune signaling, Classification model

## Abstract

**Background:**

Though potentially linked to the basic physiology of stress response we still have no clear understanding of Gulf War Illness (GWI), a debilitating condition presenting complex immune, endocrine and neurological symptoms. Here we compared male (n = 20) and female (n = 10) veterans with GWI separately against their healthy counterparts (n = 21 male, n = 9 female) as well as subjects with chronic fatigue syndrome/ myalgic encephalomyelitis (CFS/ME) (n = 12 male, n = 10 female).

**Methods:**

Subjects were assessed using a Graded eXercise Test (GXT) with blood drawn prior to exercise, at peak effort (VO2 max) and 4-hours post exercise. Using chemiluminescent imaging we measured the concentrations of IL-1a, 1b, 2, 4, 5, 6, 8, 10, 12 (p70), 13, 15, 17 and 23, IFNγ, TNFα and TNFβ in plasma samples from each phase of exercise. Linear classification models were constructed using stepwise variable selection to identify cytokine co-expression patterns characteristic of each subject group.

**Results:**

Classification accuracies in excess of 80% were obtained using between 2 and 5 cytokine markers. Common to both GWI and CFS, IL-10 and IL-23 expression contributed in an illness and time-dependent manner, accompanied in male subjects by NK and Th1 markers IL-12, IL-15, IL-2 and IFNγ. In female GWI and CFS subjects IL-10 was again identified as a delineator but this time in the context of IL-17 and Th2 markers IL-4 and IL-5. Exercise response also differed between sexes: male GWI subjects presented characteristic cytokine signatures at rest but not at peak effort whereas the opposite was true for female subjects.

**Conclusions:**

Though individual markers varied, results collectively supported involvement of the IL-23/Th17/IL-17 axis in the delineation of GWI and CFS in a sex-specific way.

## Background

An alarming number of Gulf War veterans returning from Operation Desert Storm have since been afflicted with a complex constellation of symptoms including debilitating fatigue, musculoskeletal discomfort, skin rashes, and cognitive dysfunction [1-3]. We still have no clear understanding of Gulf War Syndrome (GWS), also called Gulf War Illness (GWI), although evidence is mounting of immunological dysfunction in this population that may be potentiated by response to stress whether psychological, chemical or other. Indeed clinical presentation of GWS overlaps strongly with that of another stress-mediated illness: Chronic Fatigue Syndrome/ myalgic encephalomyelitis (CFS/ME) [4,5]. Dysregulation of the hypothalamic-pituitary-adrenal (HPA) axis has been linked to the pathophysiology of GWI [6-8] and CFS [9]. Associated pathophysiology includes hypersensitivity of normal cytokine feedback to the HPA axis [10] as well as the expected stress-induced release of neuropeptides such as NPY and its mediation of innate immune response and cortisol levels [11].

Changes within the immune signaling network have also been observed. In recent work, female adolescent CFS patients (n = 67 females; age 15 ± 1.4 years) showed a distinct immune profile when compared to acutely fatigued or non-fatigued participants, including increased production of anti-inflammatory cytokines IL-10, decreased IFNγ /IL-10 ratio and reduced production of pro-inflammatory cytokines IL-6, and TNFα in response to lipopolysaccaride (LPS) challenge *in vitro*[12]. Also suggesting an anti-inflammatory polarity in CFS, Skowera et al. (2004) [13] reported relatively high levels of intracellular IL-4 and IL-10 following *in vitro* polyclonal stimulation of whole blood from a mixed population of males and females (n = 13 males and 22 females; age 39 ± 2.1 years [19–62 years]). Bedoui, et al. (2003) [14] demonstrated that NPY, a neuropeptide found at elevated levels in female CFS patients [15] (n = 93 females; age 44 ± 0.9 years [18–60 years]), suppressed IFNγ production in Th-1 cells and increased production of IL-4 by Th2 cells. Previous work by our group in unstimulated peripheral blood plasma from a cohort of female subjects (n = 40, age 53 ± 1.8 years) also pointed to attenuated Th-1 and Th-17 immune responses in CFS with high Th-2 marker expression [16]. In contrast Moss et al. (1999) [17] reported elevated levels of TNFα in serum from CFS subjects in a mixed group of male and female subjects spanning a broad range of ages (n = 164 females and 76 males; median age 47 years [24–76 years]). Similarly Gaab et al. (2005) [18] (n = 10 male and 11 female; age 36 years [30–47 years]) observed a positive correlation between fatigue and LPS-induced *in vitro* production of TNFα in CFS and with IL-6 in both CFS and control subjects though expression levels were found to be comparable. Furthermore Carlo-Stella et al. (2006) [19] investigated cytokine gene polymorphisms and found significant differences in TNFα and IFNγ genotypes in CFS subjects suggesting that they might be genetically predisposed to differences in inflammatory response.

In contrast to CFS, studies of immune response in GWI subjects have aligned with specific exposure models and have been conducted in cohorts that were either exclusively or predominantly male. Though Rook and Zumla (1997) [20] initially postulated a Th-2 shift potentially arising from multiple vaccines, as a characteristic immune signature in this population, this was not substantiated by Zhang et al. (1999) [21]. The latter found evidence of a type I response with significant up-regulation of RNA transcript for IL-2 and IFNγ but not IL-4 nor IL-6 in a mixed population of male and female CFS veterans (n = 32 male, 11 female veterans; age 36 [20- over 50]). Similarly Skowera et al. (2004) [22] also reported a low-grade, ongoing immune activation with the characteristics of a Th-1 type response in a mixed cohort (n = 40) of male and female veterans with multi-symptom illness, in particular elevated intracellular levels of IL-2 and IFNγ as well as anti-inflammatory cytokine IL-10 following *in vitro* polyclonal stimulation of whole blood when correcting for age, sex and vaccination status. In a subsequent exploration of vaccination effects, Allen et al. (2006) [23] found an approximately equal mix of Th-1 (IFNγ and IL-2) and Th-2 (predominantly IL-13) recall response to anthrax vaccine, whereas response to plague was polarized toward Th-1 in male GW veterans (n = 17; age 39 ± 2.5 years). Along these same lines Brimacombe et al. (2002) [24] concluded that while Th-1 makers described CFS status in Gulf War veterans, a Th-2 response factor exerts an effect on cognitive function in this population. A mixed Th-1:Th-2 immune status is also consistent with our recent work in a small cohort of male subjects (n = 11, age 43 ± 2.1 years [30–55 years]) where we found higher response to PHA stimulation in GWI subjects for TNFα at rest as well as in IL-5 and IFNγ during the course of a maximal exercise challenge [25]. Indeed in a broad review of this literature and epidemiological findings, Peakman et al., (2006) [26] did not find evidence to support a dominant polarization towards Th-2 immune status alone in symptomatic GW veterans.

There is a growing body of evidence supporting a significant role for factors produced by the nervous and endocrine systems in altering immune cell function [27]. On this basis we hypothesize that lack of agreement in the above mentioned studies of cytokine signatures in these illnesses may be linked at least in part to sex-specific differences in endocrine-immune cross-talk that are also illness-specific. Preliminary work by our group using an exercise paradigm in a pilot-scale cohort of male GWI subjects (n = 10 training set, 16 validation set, age 42 ± 1.2 [33–58 years]) indicated noticeable gender and illness-specific differences in an abbreviated panel of plasma and PHA-stimulated cytokines with levels found in male CFS subjects (n = 9, age 42 ± 2.8 [28–56 years]), and female GWI subjects (n = 10, 47 ± 2.0 [38–58 years]) [28]. Indeed a large number of existing studies have been carried out in cohorts that consist of either male or female subjects. Though both sexes have been used in some of these studies, notably Skowera et al. (2004) [22], they have not for the most part been compared directly. In addition work like that of Skowera et al. (2004) [22] typically profiled a very focused subset of Th-1 and/or Th-2 cytokines. In this work we have extended the work of Skowera et al. in two ways. First we used a broader panel consisting of 16 cytokines measured in plasma. These include additional innate signals such as IL-1, 8, 12 and 15, as well as components of the IL-23/Th-17/IL-17 axis [29,30]. Not only have we included both sexes as in Skowera et al. (2004) [22], but we also compared their cytokine signatures directly using a maximal exercise paradigm to stimulate immune response for both CFS and GWI. In addition to a direct comparison of individual cytokine levels, we constructed linear classification models to identify characteristic cytokine subsets for each group. We found a recurrent theme consisting of cytokines directly or indirectly involved with Th-17 activity, i.e. IL-23, IL-17, as significant contributors to illness signature in both CFS and GWI across both sexes. This was accompanied by contributions from IL-2, IL-4, IL-10 and IL-12 in CFS and IL-5, IL-13, IL-15, IL-10 and IFNγ in GWI, many of which were only observable under challenge and during recovery. We submit that gender and illness specific differences in Th-17 modulation may constitute an important component of both CFS and GWI. Importantly, confirmation of this result would require that male and female CFS and GWI subjects be considered 4 distinct groups for observational, mechanistic, diagnostic, and treatment purposes.

## Methods

### Cohort and clinical assessment

As part of a larger ongoing study a subset of male and female subjects diagnosed with CFS/ME (n = 12 male, n = 10 female), GWI (n = 20 male, n = 10 female) and healthy but sedentary Gulf War era veterans (n = 21 male, n = 9 female) were recruited from the Miami Veterans Administration Medical Center. All subjects were comparable in age, body mass index (BMI), ethnicity and duration of illness. Subjects ranged in age between 30 and 55. Inclusion criteria for GWI was derived from Fukuda et al. (1998) [2], and consisted in identifying veterans deployed to the theater of operations between August 8, 1990 and July 31, 1991, with one or more symptoms present after 6 months from at least 2 of the following: fatigue; mood and cognitive complaints; and musculoskeletal complaints. Subjects were in good health prior to 1990, and had no current exclusionary diagnoses [31]. Medications that could have impacted immune function were excluded as were supplements. With regard to pesticides or herbicide exposure, this was an urban population and we relied on GWI registry data available to survey this aspect. The use of the Fukuda definition in GWI is supported by Collins et al. (2002) [32].

Recruitment of CFS subjects was based on the case definition of Fukuda et al. (1994) [33]. Exclusion criteria for CFS included all of those listed in the current Centers for Disease Control (CDC) CFS case definition, including the listed psychiatric exclusions, as clarified in the International CFS Working Group [31]. All CFS subjects were assessed for psychiatric diagnosis at the time of recruitment with the Composite International Diagnostic Instrument (World Health Organization, 1997) [34]. Based on this assessment, we excluded subjects with DSM IV diagnoses for psychotic or melancholic depression, panic attacks, substance dependency, or psychoses as well as any subjects currently suicidal. We also excluded subjects with Borderline or Antisocial Personality Disorder. Subjects had no history of heart disease, COPD, malignancy, or other systemic disorders that would be exclusionary, as clarified by Reeves et al. (2003) [31]. Importantly, care was taken to assess female subjects at the same phase of the menstrual cycle, namely in the follicular phase. This was self-reported by subjects and based on time from end of last cycle. A summary description of the subject subsets is listed in Additional file 1: Table S1.

### Exercise challenge

Immune response was stimulated with a standard Graded eXercise Test (GXT) using a Vmax Spectra 29c Cardiopulmonary Exercise Testing Instrument, Sensor-Medics Ergoline 800 fully automated cycle ergometer, and SensorMedics Marquette MAX 1 Sress ECG. According to the McArdle protocol [35] subjects pedaled at an initial output of 60 watts for 2 minutes, followed by an increase of 30 watts every 2 minutes until the subject reached: 1) a plateau in maximal oxygen consumption (VO2); 2) a respiratory exchange ratio >1.15; or 3) the subject stopped the test. A first blood draw was conducted prior to exercise following a 30-minute rest. Second and third blood draws were conducted upon reaching peak effort (VO2 max) and at 4-hours post exercise respectively. Summary statistics describing the exercise capacity in terms of the weight-adjusted maximum VO2 measured in L/min/kg are presented for each group in Additional file 1: Table S1. Results indicate a decline in the average maximum VO2 achievable with healthy controls performing best and CFS subjects faring the worst. This trend achieved statistical significance in the male subjects (p = 0.04) with healthy male subjects performing better than both illness groups. In light of this finding we suggest that results presented here be interpreted as immune response at maximum perceived exertion but not necessarily at equivalent exercise intensity. We consider reduced exercise capacity to be another symptom of GWI and CFS. The characteristic immune response patterns measured at maximum perceived exertion capture this implicitly as well as a host of other more discriminating features of both illnesses.

### Ethics statement

All subjects signed an informed consent approved by the Institutional Review Board of the University of Miami and the Miami Veterans Affairs Medical Center. Ethics review and approval for data analysis was also obtained by the IRB of the University of Alberta.

### Laboratory measurements

Plasma was separated within 2 hours of collection and stored at −80°C until assayed. We measured 16 cytokines in plasma using Quansys reagents and instrument (Quansys Biosciences, Logan, Utah) in the same way as reported previously in a larger cohort of CFS subjects with unknown etiology and illness trigger [16,36]. The Quansys Imager, driven by an 8.4 megapixel Canon 20D digital SLR camera, supports 96 well plate based chemiluminescent imaging. The Q-Plex™ Human Cytokine - Screen (16-plex) is a quantitative enzyme-linked immunoabsorbent assay (ELISA)-based test, where sixteen distinct capture antibodies have been absorbed to each well of a 96-well plate in a defined array. The range of the cytokine concentrations used in the standard calibration samples were adjusted for each cytokine along with sample exposure time to provide the most reliable comparison possible of GWI and CFS patients with controls across the range of cytokine concentrations known and expected in plasma. We used a second order (k = 2) polynomial regression model: Y_p_ = b_0_ + b_1_X_1_ + b_2_X_2_.... + b_k_X_k_, as a calibration curve, with intercept b_0_, regression coefficients b_1_ to b_k_ for each degree and where Y_p_ was the predicted concentration. The standard sample concentrations used to establish these calibration curves for each cytokine as well as the detection limits for this assay have been described in detail in previous work by our group [16]. In brief, these support an average coefficient of variability (CV) of 0.20 for inter-assay comparisons and a value of 0.09 for intra-assay repeatability.

### Statistical analyses

To assess the significance of differences in the mean expression of individual cytokines separating each subject group we used a standard parametric t test after performing a logarithmic (log2) transformation as well as a non-parametric Wilcoxon ranksum test. The latter was used as a confirmatory measure for the sake of completeness and to assess the adequacy of the log2 transform in restoring normality to the distribution of values. As an extension of this single-marker univariate analysis, the use of cytokines in combination with one another was also considered. This was done by constructing linear discriminant classification models that used multiple cytokines in concert to distinguish individuals in each group. Cytokines are highly co-expressed as a result of the feedback and feed-forward regulatory mechanisms they support [37]. Unfortunately traditional ordinary least squares regression performs poorly when correlated or collinear terms are used together due to variance inflation [38]. Two complementary methods were used to address this issue and provide the most stable regression models possible: the first is based on truncation of highly correlated terms whereas the second is based on the creation of cytokine constructs that are minimally correlated. Based on truncation of terms, the stepwise method mitigates the effect of cross-correlation between cytokines by favoring the identification of subsets of cytokines that are minimally redundant [39]. According to this algorithm, model terms were selected sequentially based on their respective partial-F test values. Cytokines with a null probability p(partial F) < 0.05 were selected for recruitment into the regression model while those currently in the model but showing a revised p (partial F) > 0.10 were removed. In the resulting model an observed row x from the sample array is classified into group I rather than group J if 0 < B_0_ + x*B, where the coefficient vector B and intercept vector B_0_ are estimated from the data.

In some cases truncation of terms proved too severe and the complementary approach of reconciling overlapping expression patterns was used. For example, in the case of female CFS subjects the constraint of minimal colinearity, or redundancy between markers, that is implicit to the stepwise algorithm resulted in poor classifier performance. As a result, the effects of relaxing this constraint were explored using an all-possible-subsets selection method and a reconciliation of terms based on a correction of covariance matrix structure. In this case random subsets of 5 cytokines were selected from all 48 candidate biomarkers (16 cytokines at 3 time points) and evaluated using a linear classification model as described above. This was repeated over 500 iterations. Individual cytokines were then ranked according to the frequency with which they were selected in classification models that supported an assignment accuracy of at least 80%. In each candidate model deviations from mutual independence between markers was corrected using a diagonal covariance matrix estimate [40].

All cytokine concentrations were log2 transformed and normalized to have a mean value of 0 and a variance of 1.0. Missing values were imputed using a non-zero value of 0.0001 in order to make maximal use of all profiles in the regression calculations. Leave-one-out cross validation was used as a measure of the predictive stability of each model. According to this strategy class assignments for each subject are performed based on models constructed in the absence of that particular subject’s cytokine profile. All classifiers were evaluated based on their leave-one-out cross-validation performance rather than their ability to fit the training data. Performance measures used include overall accuracy (correctly classified samples / total classified samples) in assigning subjects to their proper diagnostic group, sensitivity (correctly classified positive / true positive samples), specificity (correctly classified negative / true negative samples), positive predictive (correctly classified positive / positive classified samples) and negative predictive (correctly classified negative / negative classified samples) values.

All calculations described above were performed using the SPSS Statistics software platform version 19 (IBM, SPSS Inc., Chicago, IL) as well as the *classify, classperf, and stepwisefit* functions available in the MatLab Statistics Toolbox and the MatLab Bioinformatics Toolbox (The MathWorks, Inc., Natick, MA).

## Results

### Gender-specific differences in immune signatures among healthy controls

In order to assess the importance of sex-specific immune response to exercise a comparison of cytokine expression profiles in male and female subjects was conducted in the healthy control group. In a first analysis, the log 2 transformed expression levels of individual cytokines were compared using a standard t test and a non-parametric Wilcoxon test at each phase of exercise. Results presented in Additional file 1: Table S2 show significant differences in cytokine expression across sexes, namely elevated IL-23 in healthy female subjects at all time points (p ≤ 0.01) as well as elevated IL-12 expression post-exercise (p = 0.03). A trend towards higher IL-5 levels at peak effort in male subjects (p = 0.08) was also observed but this did not achieve statistical significance. Cytokines such as these are not expressed independently of one another but instead their patterns arise as a result of one or several basic immune processes acting in concert. In an attempt to explore concurrent immune processes a step-wise variable selection algorithm was used to select subsets of cytokines which acting in combination might further support the separation of male and female subjects in both illness and healthy control groups. The results of this modeling only emphasized the importance of the immunological differences between sexes.

**Figure 1:**
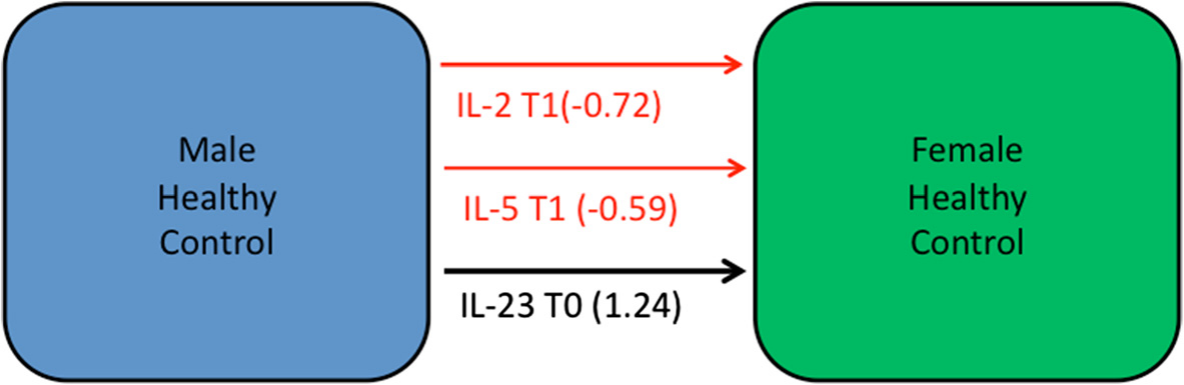
**Gender differences in immune signatures among healthy controls.** Immune cytokine expression of male and female healthy controls was compared using a step-wise method across all time points (rest, peak activity, and recovery). Red arrows indicate a negative contribution of a particular cytokine in female healthy controls compared to males and vice versa for black arrows. Arrow line thickness is proportional to the magnitude of the standardized canonical coefficients. Changes in IL-23 were a dominant factor at all time points, in particular when cast against concurrent levels of IL-2 and IL-5 expression under challenge.

The performance of classification models separating male from female subjects within the healthy control, CFS and GWI groups respectively is summarized in Table 1 (Figure 1). Results indicate that male and female subjects in the healthy control group can be readily distinguished at rest (T0) by as few as 2 cytokines, namely IL-2 and IL-23, with a specificity of 78% and sensitivity of 90%. The use of an exercise challenge only improves upon this, with levels of IL-23 at rest (T0) in combination with IL-2 and IL-5 at peak effort supporting 89% specificity with 90% sensitivity. Differences in cytokine profile between the sexes remain significant in both GWI and CFS but with somewhat lower specificity in GWI (70%) and sensitivity in CFS (67%). Of particular interest, no cytokines out of the 16 measured could distinguish between the sexes in GWI subjects at rest. Only under exercise challenge could male GWI subjects be distinguished from their female counterparts on the basis of cytokine expression. Collectively these differences in immune response to exercise strongly support a stratification of the cohort on the basis of sex prior to any analysis of illness-specific cytokine signatures.

**Table 1:** Linear classification models separating male from female subjects within each diagnostic group

	**Model**	**Stand. coeff.**	**Assigned male**	**Assigned female**	**Total**		**Accuracy**	**NPV**	**PPV**	**Specificity**	**Sensitivity**
*Healthy Ctrl*											
mHC vs fHC at T0	Il-2 T0	−0.81	19	2	21	True male	0.87	0.90	0.78	0.90	0.78
	Il-23 T0	1.14	2	7	9	True Female					
mHC vs fHC at T0-T2	Il-2 T1	−0.72	19	2	21	True male	0.90	0.95	0.80	0.90	0.89
	Il-5 T1	−0.59	1	8	9	True Female					
	Il-23 T0	1.24									
*CFS*											
mCFS vs fCFS at T0	Il-8 T0	1.00	7	5	12	True male	0.68	0.78	0.62	0.58	0.80
			2	8	10	True Female					
mCFS vs fCFS at T0-T2	TNFb T2	−0.91	8	4	12	True male	0.77	0.89	0.69	0.67	0.90
	Il-6 T1	−0.83	1	9	10	True Female					
	Il-8 T0	1.75									
*GWI*											
mGWI vs fGWI at T0	*NA*						NA	NA	NA	NA	NA
mGWI vs fGWI at T0-T2	Il-2 T2	−1.51	19	1	20	True male	0.87	0.86	0.88	0.95	0.70
	IL-1a T0	0.60	3	7	10	True Female					
	IL-1a T1	−1.08									
	Il-6 T1	0.84									
	Il-10 T1	1.36									

Performance values correspond to leave-one-out cross validation results.

### Characteristic cytokine signatures of illness in male subjects

**Figure 2:**
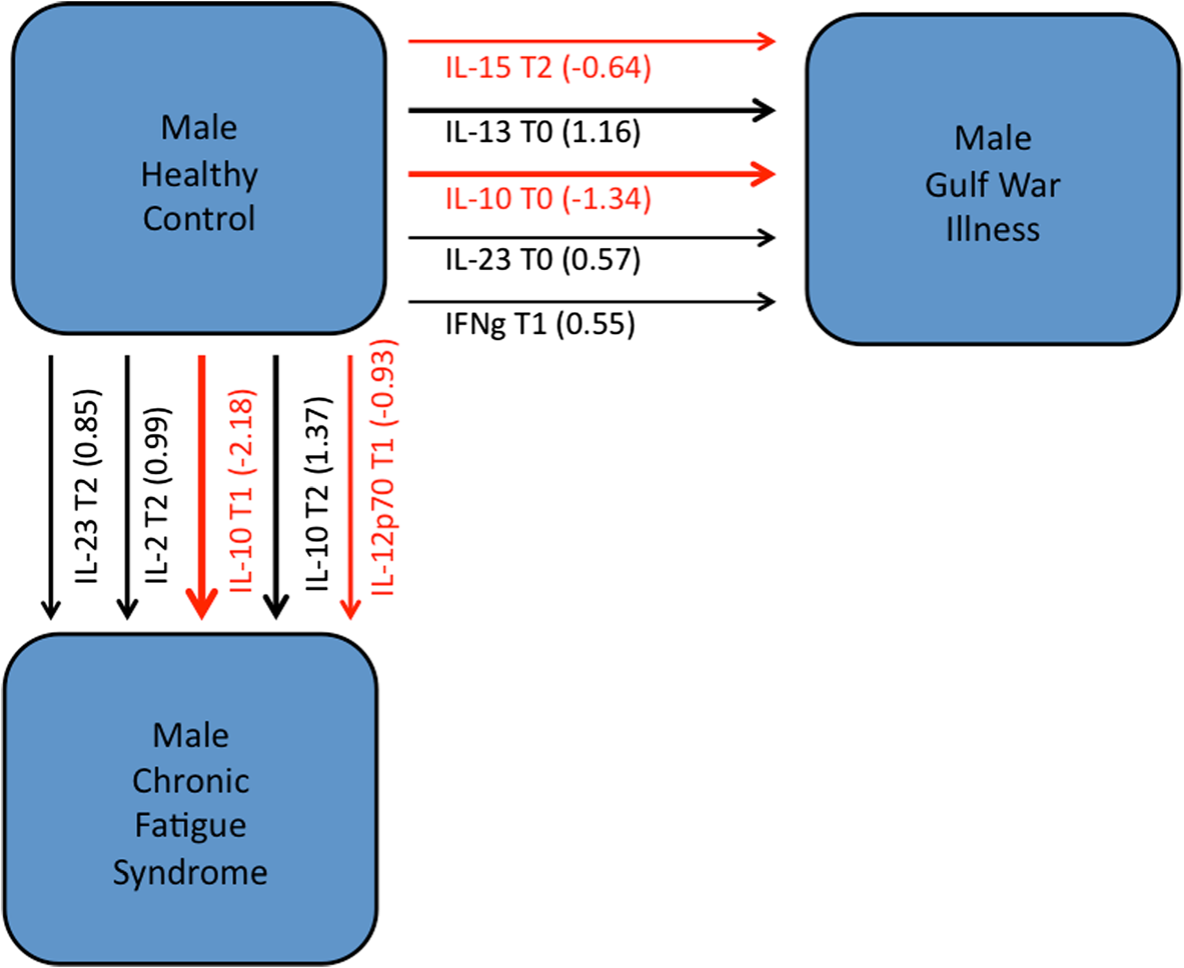
**Cytokine signatures separating illness groups in male subjects.** Immune cytokine expression patterns separating of male GWI, CFS and healthy control subjects identified using a step-wise selection method across all time points (rest, peak activity, and recovery). Red arrows indicate a negative contribution, vice versa for black arrows. Arrow line thickness is proportional to the magnitude of standardized canonical coefficients. NK cell promoters (IL-12 or 15) are observed in both illnesses, as are IL-10 and 23. Stronger IL-2 expression was characteristic of CFS while stronger IL-13 contribution was indicative of GWI (Table 2 and Additional file 2: Table S5).

Once again the expression levels of individual cytokines were compared using the standard t and Wilcoxon ranksum tests. Results are presented for male subjects in Additional file 1: Table S3. These suggest that levels of IL-8 and IL-13 (p = 0.04, 0.03) were significantly elevated in male GWI subjects at rest, however this was not supported by the Wilcoxon test. In contrast, both parametric and non-parametric tests indicate that male CFS subjects expressed significantly higher levels of IL-2; both at peak effort and at 4 hours post exercise (p = 0.03, 0.02). These subjects also expressed significantly higher levels of IL-23 both prior to and following exercise (p = 0.01, 0.01) but not at peak effort. Using the same approach as before, cytokine subsets were identified that best supported a linear model for the classification of male subjects at each phase of exercise as well as over the entire challenge. These results are described in Table 2 (Figure 2). While healthy male subjects could be distinguished from male GWI subjects at rest (T0) and during recovery (T2), these groups could not be distinguished on the basis of cytokine signatures at peak activity (T1). At rest (T0), differences in IL-13 in male GWI subjects were best considered in the context of IL10, and IL-23, two cytokines that did not differ significantly in expression level. Used together as an illness signature for GWI this combination of cytokines delivered a predicted specificity of 76% and a sensitivity of 70% (PPV = 74%; NPV = 73%) in a leave-one-out cross-validation scenario. At recovery (T2), the classification of male GWI subjects was again supported by expression of IL-13 in the context of IL-10 and IL-23. Levels of IL-1b expression also contributed significantly in providing a contextual basis for changes in IL-13. These four cytokines used in combination supported higher sensitivity (80%) than the model identified at rest but with a somewhat lower specificity (67%) (PPV = 70%; NPV = 78%). When aggregating cytokine profiles across all three time points the subset of IL-13, 10 and 23 measured at rest were again retained and additional context was recruited in the form IFN-γ at peak effort and IL-15 post-exercise. Used together, these cytokines measured across all three phases of exercise delivered a sensitivity of 85% and a specificity of 81% in cross-validation (PPV = 81%; NPV = 85%).

**Table 2:** Linear classification models separating healthy male subjects from male subjects with GWI or CFS

	**Model**	**Stand. coeff.**	**Assigned HC**	**Assigned GWI or CFS**	**Total**		**Accuracy**	**NPV**	**PPV**	**Specificity**	**Sensitivity**
*Male GWI Subjects*											
GWI vs HC males at T0	Il-10 T0	−1.32	16	5	21	True HC male	0.73	0.73	0.74	0.76	0.70
	Il-13 T0	1.23	6	14	20	True GWI male					
	Il-23 T0	0.53									
GWI vs HC males at T1	*NA*					True HC male	NA	NA	NA	NA	NA
						True GWI male					
GWI vs HC males at T2	Il-1b T2	−0.59	14	7	21	True HC male	0.73	0.78	0.70	0.67	0.80
	Il-10 T2	−1.10	4	16	20	True GWI male					
	Il-13 T2	1.02									
	Il-23 T2	0.62									
											
GWI vs HC males at T0-T2	Il-15 T2	−0.64	17	4	21	True HC male	0.83	0.85	0.81	0.81	0.85
	Il-10 T0	−1.34	3	17	20	True GWI male					
	Il-13 T0	1.16									
	Il-23 T0	0.57									
	IFNg T1	0.55									
*Male CFS Subjects*											
CFS vs HC males at T0	Il-23 T0	1.00	16	5	21	True HC male	0.76	0.84	0.64	0.76	0.75
			3	9	12	True CFS male					
CFS vs HC males at T1	Il-2 T1	1.84	16	5	21	True HC male	0.82	0.94	0.69	0.76	0.92
	Il-6 T1	−0.66	1	11	12	True CFS male					
	Il-10 T1	−0.75									
	Il-15 T1	−0.66									
											
CFS vs HC males at T2	Il-23 T2	1.00	16	5	21	True HC male	0.73	0.80	0.62	0.76	0.67
			4	8	12	True CFS male					
CFS vs HC males at T0-T2	Il-2 T2	0.99	18	3	21	True HC male	0.85	0.90	0.77	0.86	0.83
	Il-10 T2	1.37	2	10	12	True CFS male					
	Il-23 T2	0.85									
	Il-10 T1	−2.18									
	Il-12p70 T1	−0.93									

Performance values correspond to leave-one-out cross validation results.

Using a similar approach we attempted to isolate a characteristic subset of cytokines in the smaller illness group of male CFS subjects. Reflecting a shared trend with GWI, a linear classification model based solely on IL-23 levels at rest distinguished between male CFS and control subjects with a predicted classification sensitivity of 75% and a specificity of 76% in leave-one-out cross-validation. Though false negative predictions were limited (NPV = 84%), use of this marker alone produced a high number of false positive assignments (PPV = 64%). An identical model was identified in the recovery phase with very similar classification performance. At peak effort, differences in IL-2 expression were cast in the relative context of concurrent levels of IL-6, 10 and 15. Taken together these produced a predicted classification sensitivity of 92% but specificity remained unchanged at 76%. Accordingly the negative predictive value improved (NPV = 94%) but only marginal change in positive predictive rate was produced (PPV = 69%). Only by accounting for all exercise phases was it possible to produce a more balanced classification performance. Using IL-2, 10, 12 and 23 measured selectively at rest (T1) and post-exercise (T2) noticeably improved the classification performance albeit in a small subject group (sensitivity = 83%; specificity = 86%; PPV = 77%; NPV = 90%). This emphasized the importance of an exercise paradigm in investigating CFS in particular. Common to both GWI and CFS illness signatures were the direct or indirect contributions of IL-10 and IL-23 expression though these occurred at very different times. While levels measured at rest supported an illness signature in GWI, their impact in CFS was only observable during and after exercise, again emphasizing the importance of a challenge and response timeline in distinguishing these illnesses.

### Cytokine signatures of illness in female subjects

**Figure 3:**
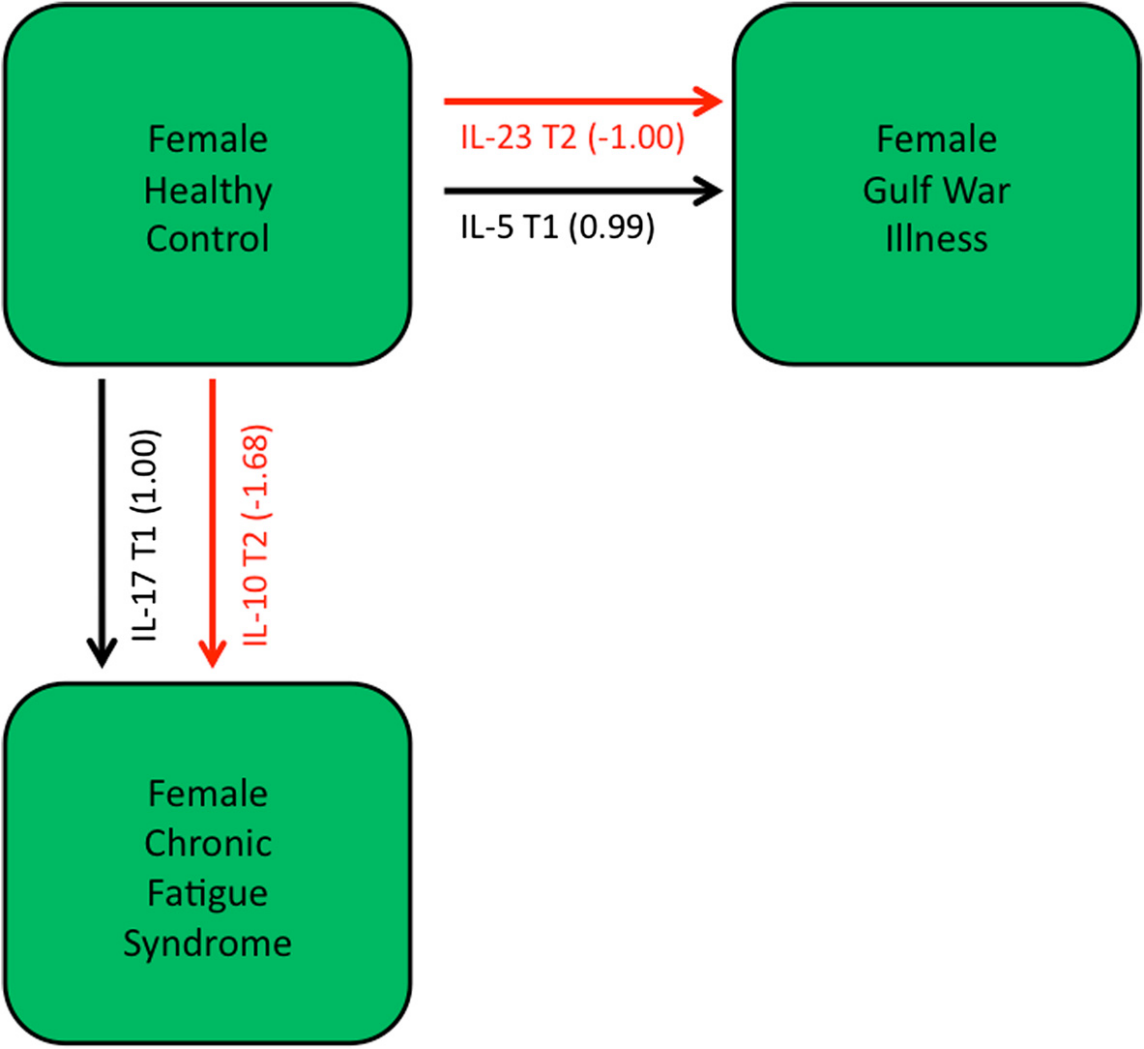
**Cytokine signatures separating illness groups in female subjects.** Immune cytokine expression patterns separating of female GWI, CFS and healthy control subjects identified using a step-wise selection method across all time points (rest, peak activity, and recovery). Red arrows indicate a negative contribution, vice versa for black arrows. Arrow line thickness is proportional to the magnitude of standardized canonical coefficients. While decreased IL-23 expression in the context of increased IL-5 levels provided strong separation of GWI from healthy controls the signal for CFS was much weaker and required relaxation of the constraints on cross-correlation between cytokines.

In a similar analysis of female subjects we were unable to identify any cytokines that, when measured at rest, supported a characteristic signature for GWI. As in their male counterparts, levels of IL-8 were significantly elevated in female GWI subjects. However unlike the former, this was only detectable at peak effort (T1) (p = 0.04) as were elevated levels of IL-5 (p = 0.03) (Additional file 1: Table S4). Also found were significantly lower levels of IL-23 at recovery (T2) (p = 0.03). This cytokine was only marginally increased in male subjects at the same phase of exercise (Additional file 1: Table S3). Levels of IL-1a measured at recovery, and IL-10 measured at peak effort, were also found significantly lower in GWI female subjects based on the Wilcoxon ranksum test (p = 0.02, 0.03). Identification of a linear classification model for female GWI subjects cast increased IL-5 expression in the context of IL-17 levels at peak effort (T1). Used in combination these two cytokines measured at peak effort supported classification of female GWI relative to control subjects with a sensitivity of 80% and a specificity of 89% (PPV = 89%; NPV = 80%) in cross-validation, albeit in a smaller pilot cohort (n = 10). At recovery, the significantly lower expression of IL-23 alone was sufficient to distinguish female GWI from control with a predicted classification sensitivity of 70% and a specificity of 89% (PPV = 88%; NPV = 73%). This did not improve noticeably when allowing the selection of IL-5 at peak effort and IL-23 post-exercise from all phases of exercise (Figure 3). It is interesting to note that contrary to the models identified for male GWI, there is a very limited contribution of contextual markers in the classification models for female subjects. Indeed, only those cytokines showing significant differences in expression were selected.

In comparison, it was very difficult to find any strong differences in cytokine expression separating female CFS subjects from healthy control. Indeed, only decreased expression of IL-4 (p = 0.02) and increased expression of IL-1a (Wilcoxon p = 0.04) measured at peak effort in female CFS subjects reached statistical significance in this data (Additional file 1: Table S4). Nor did casting this difference in the context of other cytokines create a more robust illness signature. In all classification models, very high false positive rates were produced in leave-one-out cross-validation regardless of the time point considered (Table 3). Positive predictive values were less than 65% with specificity values below 50%. It is important to remember that stepwise selection applies strict constraints to encourage the recruitment of mutually independent markers. In an alternative approach, we applied an all-possible-subsets selection with a multivariate correction to relax this constraint and allow the inclusion of cytokines that might be more closely co-expressed. This more exhaustive search identified IL-17 at peak effort (T1) and IL-10 during recovery (T2) as two of the most frequently selected markers in 500 random subsets of 5 cytokines. Using these two markers together and applying a canonical correction for co-expression supported a classification specificity of 89%, at a sensitivity of 70% (PPV of 88%) (Table 3). Inclusion of IL-5 measured at T2, the third ranking marker, supported a complete separation of female CFS subjects from their healthy counterparts. An examination of the correlation structure confirmed that expression of IL-10 at T2 and IL-17 at T1 and T2 were significantly correlated (p = 0.04, 0.05) making them undesirable candidates in the initial stepwise selection scheme.

**Table 3:** Linear classification models separating healthy female subjects from female subjects with GWI or CFS

	**Model**	**Stand. coeff.**	**Assigned HC**	**Assigned GWI or CFS**	**Total**		**Accuracy**	**NPV**	**PPV**	**Specificity**	**Sensitivity**
*Female GWI Subjects*											
GWI vs HC females at T0	*NA*					True HC female	NA	NA	NA	NA	NA
						True GWI female					
GWI vs HC females at T1	Il-5 T1	1.20	8	1	9	True HC female	0.84	0.80	0.89	0.89	0.80
	Il-17 T1	−1.03	2	8	10	True GWI female					
GWI vs HC females at T2	Il-23 T2	−1.00	8	1	9	True HC female	0.79	0.73	0.88	0.89	0.70
			3	7	10	True GWI female					
GWI vs HC females at T0-T2	Il-23 T2	−1.00	8	1	9	True HC female	0.84	0.80	0.89	0.89	0.80
	Il-5 T1	0.99	2	8	10	True GWI female					
*Female CFS Subjects*											
CFS vs HC females at T0	Il-4 T0	−0.84	5	4	9	True HC female	0.74	0.83	0.69	0.56	0.90
	Il-17 T0	0.77	1	9	10	True CFS female					
CFS vs HC females at T1	Il-2 T1	−1.00	4	5	9	True HC female	0.63	0.67	0.62	0.44	0.80
			2	8	10	True CFS female					
CFS vs HC females at T2	Il-2 T2	−1.00	3	6	9	True HC female	0.68	1.00	0.63	0.33	1.00
			0	10	10	True CFS female					
CFS vs HC females at T0-T2	Il-2 T1	−1.00	4	5	9	True HC female	0.68	0.80	0.64	0.44	0.90
			1	9	10	True CFS female					
** All possible subsets											
CFS vs HC females at T0-T2	IL-17_T1	1.00	8	1	9	True HC female	0.79	0.73	0.88	0.89	0.70
	IL-10_T2	−1.68	3	7	10	True CFS female					

Performance values correspond to leave-one-out cross validation results.

When comparing female GWI and CFS immune signatures directly, highly significant differences were observed at all three time points. Significantly lower levels of IL-4 (p = 0.04, 0.02, 0.01) and IL-8 (p = 0.02, 0.00, 0.01) were observed in female CFS subjects compared to their GWI counterparts at all three phases of exercise. Similarly decreased levels of IL-5 were observed at rest and peak effort in CFS compared to GWI in female subjects (p = 0.01, 0.01). Lower levels of TNFα were also found during recovery using the ranksum test (p = 0.02). These same cytokines were expressed at intermediate levels in healthy control subjects suggesting a reversal of polarity in expression profile for CFS compared to GWI in female subjects only. In keeping with these differences, female GWI and CFS groups separated almost perfectly on the basis of IL-5 and IL-1b levels alone measured at rest (Additional file 2: Table S5). This pattern of opposites was not observed in male subjects where cytokine levels in CFS and GWI typically trended in the same direction in comparison to control. As a result, these groups could only be separated when the requirement of mutual independence was relaxed. Indeed when allowing for selection and reconciliation of IL-1β and TNFβ co-expression at rest, two highly correlated markers (r = 0.81, p < 0.01 in CFS), we obtained an assignment accuracy of 66% for male GWI versus male CFS subjects. These groups separated almost completely with the addition of IL-2 and IL-6 co-expression at peak effort (Additional file 2: Table S5).

## Discussion

GWI and CFS remain poorly understood illnesses with complex etiology. Though they show considerable overlap in clinical presentation there is mounting evidence supporting the involvement of characteristic immune and endocrine dysfunction in each illness. In this work we compared these sister illnesses and their cytokine expression profiles in both male and female subjects separately to explore the role of endocrine-mediated modulation of immune response. As persistent and disabling fatigue is a key presenting symptom in both illnesses, a graded exercise challenge was used to stimulate immune response and amplify cytokine expression in a clinically relevant way. Several cytokines are recognized as exercise-responsive or metabolically active in healthy individuals. These myokines include IL-1ra, 6, 8, 10, 15 and TNF-α [41,42]. In this work IL-10 and IL-15 appear as illness markers in male subjects with expression levels suggesting a delayed or compromised anti-inflammatory response to exercise. The same would appear true of exercise response in female CFS subjects. Interestingly significant differences in the immune response to exercise between male and female populations were immediately observed even in the healthy control group. Healthy female subjects could be distinguished from their male counterparts by significantly elevated expression of IL-12 and IL-23. While male GWI subjects could be distinguished from control on the basis of cytokine expression at rest but not at peak effort, the opposite was true for female subjects. Moreover, female GWI and CFS subjects could be separated almost perfectly on the basis of IL-1b and IL-5 levels alone, albeit in a small pilot cohort (n = 10). Indeed, cytokine expression would typically trend lower than control in female CFS and higher than control in female GWI, reaching statistical significance for levels of IL-4, 5 and 8. In contrast, male GWI could not be distinguished easily from their CFS counterparts on the basis of minimally overlapping cytokine markers as these typically trended in the same direction compared to control. Separation of these subjects was achieved in previous work by our group [28] but this work made extensive use of cytokine response measured in supernatants isolated and treated *in vitro* with the mitogen phytohaemagglutinin (PHA), a stimulant of T cell activity. The current work would suggest these groups may share several features of cytokine co-expression in plasma and that the patterns and levels obtained in response to *in vitro* stimulation with a specific mitogen may be required to provide the resolution necessary to distinguish one set from the other unequivocally. Moreover, as in this previous analysis it may also be necessary to reconcile partially correlated cytokines and use these in conjunction with one another instead of truncating in order to converge on a set of minimally overlapping cytokines. This observation was further confirmed in our analysis of female CFS subjects. Separation of these subjects from healthy control improved dramatically when the requirement for mutual independence was relaxed and statistical corrections were made to include and reconcile the co-expressed markers IL-17 at peak effort and IL-10 at recovery. Likewise male CFS and GWI subjects could be separated almost completely when adjusting for the co-expression of TNFβ, IL-1β, 2 and 6 at rest and peak effort.

These differences not withstanding, in looking across all subject groups we found a recurrent theme consisting of cytokines directly or indirectly involved with Th17 activity [30]. For example, IL-23 was a distinguishing feature of sex in healthy subjects. It was also a significant contributor to illness signature in both CFS and GWI, along with IL-17 across both sexes. In one subset or another, this was accompanied by contributions from IL-1b, IL-2 and IFNγ among others. In addition IL-6 and IL-8 contributed to differences between male and female subjects within each illness group. It is interesting to note that most of these cytokines were not differentially expressed across groups even though they contributed significantly to the classification of these subjects. IL-1, 6 and 23 are known inducers of Th17 response, which in turn is a producer of IFNγ [30]. Conversely IL-2 is generally known as a Th17 antagonist [43]. Indeed the expression of these cytokines is not as disparate as it may seem. For example, Liu et al. (2007) [44] describe a mechanism by which IL-1 induces the production of IL-23 via NF-kappa B activation, which in turn promotes the production of IL-6 and 8 in human fibroblast-like synoviocytes from rheumatoid arthritis patients. IL-10 was also selected across illnesses, particularly in male subjects, as an important contextual element for IL-23 expression. Work by Verreck et al. (2004) [29] has implicated the interplay between IL-23-producing type 1 macrophages and IL-10-producing type 2 macrophages in modulating immune response to mycobacteria.

Overlap between IL-23 as an illness marker and its contribution (along with family member IL-12) as a marker of gender-specific response to exercise suggests significant involvement of the sex hormone axis in modulating the IL-23/Th17/IL-17 immune response. For example there is evidence that progesterone may suppress Th1 and Th17 pathway activity and induce an anti-inflammatory response when present at pregnancy levels [45]. Conversely progesterone has been shown to enhance Th2 and T regulatory (Treg) responses. In the current analysis the appearance of IL-4 and IL-5 as markers of GWI and CFS in the female subset further supports possible involvement of a sex hormones in modulating the balance between Th2 and Th17 response. Studies in human cell cultures and in mice have shown that human T cells exhibit a sex-specific difference in the production of IFNγ and IL-17 driven by androgen status and possibly the expression of peroxisome proliferator activated receptors PPARα and PPARγ [46]. In fact, in animal models the disappearance of normal sex-driven differences in the gene regulatory network modulating Th17 immune response has been implicated recently in supporting an increased susceptibility to rheumatoid arthritis (RA) [47]. Collectively these works and the analysis presented here suggest that imbalance in the sex hormone regulation of Th17 immune response may be a shared component of these illnesses.

In GWI specifically, recent work has pointed to disruption of normal cholinergic regulation of immune function as a possible result of exposure to battlefield agents [48,49]. For example, mRNA levels of cytokines IL-6, IL-17 and IL-13 among others have been shown to respond readily to organophosphate exposure [50]. Acetylcholine (ACh) is known to regulate T cell function by acting on the nicotinic (nAChR) and muscarinic (mAChRs) cholinergic receptors [51]. In recent work, Qian et al. (2011) [52] demonstrated in culture that nicotinergic stimulation of CD4+/CD62L(L-selectin) + T cells upregulated IFNγ and down-regulated IL-17 secretion, whereas the muscarinic stimulation enhanced IL-10 and IL-17 while inhibiting INFγ secretion. Muscarinic acetylcholine signaling has also been found to promote IL-8 release via PKC, ERK1/2 and NF-ΚB pathways [53]. Similarly nicotinic receptor (nAChR) antagonists, ERK1/2 inhibitors or intracellular calcium chelators reduced IL-8 secretion by human epithelial cells [54]. Based on this finding, persistent cholinergic stimulation would support the increased levels of IL-8 observed here in both male and female GWI subjects. Increased nicotanergic activation would support an ACh-driven down-regulation of Th17 response evidenced by decreased IL-23 in female GWI subjects. Elevated IL-5 levels in this group would further support the suppression of IL-17 response as part of a sex hormone mediated shift towards a Th2 status. The absence of this added sex-specific driver of Th2 polarization might explain the more subdued trend towards Th17 suppression observed in male GWI subjects. It is important to note that the above-mentioned mechanisms were typically studied under specific experimental conditions whereas the immune signatures reported in this work were obtained in whole blood from human subjects. In order to obtain a more detailed understanding of the precise mechanisms that underlie these cytokine profiles it will be important to cast these in the context of the corresponding immune cell demographics using flow cytometric methods [55].

Though with some illness-specific variations, a continued trend away from Th2 polarity is observed in male CFS subjects with these subjects showing considerable overlap in cytokine expression with their GWI counterparts. We also found a decrease in Th2 polarity when comparing levels of IL-4 and IL-5 in female CFS with those in female GWI. In our earlier work with a much larger cohort of female CFS subjects (n = 40; mean age 53 ± 1.8 years) [16], we had found elevated levels of Th2 markers in female CFS subjects when compared to healthy controls. As discussed above, a potentially important contributor to this may be menopausal status since sex hormones can be important modulators of Th2 polarization [45,56] and age has been shown to be an important contributor to increased IFNγ response in women [57]. In comparison, the opposite trend in IFNγ response with age is typically true in men and occurs over a more narrow range. It is important to note therefore that this previous work with female CFS subjects was biased towards a post-menopausal population (n = 40; mean age 53 ± 1.8) whereas the smaller cohort used in this analysis would be more adequately described as peri-menopausal (n = 10; mean age 43 ± 2.8). Indeed when we compared healthy control groups across these studies we found significantly lower expression of IL-4 in the older control group (p = 0.003) while IL-4 levels in both the older and younger CFS groups were comparable (p = 0.49) (Additional file 3: Figure S1). Certainly the reduced size of the current cohort, in particular the number of female subjects, may have limited the statistical resolution for detecting differences in the expression levels of individual cytokines. However, the compatibility of healthy control groups should almost certainly be considered when comparing immune signatures across studies especially in women, as these control populations constitute a moving endocrine-immune benchmark. In addition to sex hormones, stress and growth hormones produced along the hypothalamic-pituitary-adrenal (HPA) and hypothalamic-pituitary-thyroidal (HPT) axes are also important immune modulators [58,59]. Moreover, dysfunction of these axes has also been documented in these illnesses [6,60]. Although in this study the immune-modulatory effects of these hormones were implicit to the cytokine signatures, their direct measurement would undoubtedly reduce unexplained variations in immune signature and potentially improve the resolution of a diagnostic panel for these illnesses.

These limitations notwithstanding, certain preliminary observations regarding the immune processes involved in these illnesses may still be made. In particular, it was interesting to note that the IL-23/Th17/IL-17 axis appears differentially modulated across sexes in healthy individuals and that different markers of this same axis appear to contribute to the delineation of GWI and CFS in a sex and illness-specific way. Our work across multiple cohorts has shown this theme of Th17 dysfunction to be highly conserved even though individual markers may not be. This work also suggests that more robust cytokine signatures may be obtained by accounting for sex-specific differences in endocrine-immune crosstalk and that future studies should consider male and female CFS and GWI subjects as 4 distinct groups for observational, mechanistic, diagnostic, and potentially treatment purposes as well.

## Conclusions

In summary even though additional study in larger cohorts is needed to further substantiate the diagnostic potential of these individual biomarkers, certain themes emerge nonetheless. In particular, we consistently found the expression of cytokines directly or indirectly involved with Th17 activity as significant contributors to illness signature in both CFS and GWI, across both sexes. However, trends in expression levels relative to control subjects varied between groups, as did the choice of additional markers. These trends were more subtle and co-expression patterns more tightly integrated in CFS, in particular in female subjects. In addition, while male GWI subjects could be distinguished at rest but not at peak effort, the opposite was true for female subjects. These observations further reinforce the need for stratification according to gender as well as the importance of an exercise challenge in these studies. Indeed, a comparison with our previous study of another CFS cohort suggested that female subjects should be stratified further with respect to age and menopausal status. This observation together with the gender differences found in this work support an important role for endocrine modulation of immunity in these illnesses and argue in favor of stratification of cohorts into separate groups in each illness according to sex.

## Competing interests

The authors declare that they have no competing interests.

## Authors' contributions

Conceived and designed the experiments: MAF NGK GB. Performed the experiments: MAF, NGK, ZB, SR, FC, CS. Analyzed the data: ALS, GB, HF. Contributed reagents/materials/analysis tools: MAF, NGK, GB. Wrote the paper: ALS, GB, HF, FC, CS, NGK, MAF. All authors read and approved the final manuscript.

## Supplementary Material

Additional file 1: Table S1 Cohort description of mean values (std. error) with the ANOVA null probability for significance of the group effect. **Table S2.** Cytokine expression values, mean (std. error) at rest (T0), peak effort (T1) and post-exercise (T2) in healthy male and female subjects. Null probability values are based on a two-tailed t test performed on Log2 transformed data, and Wilcoxon ranksum on untranformed data. **Table S3.** Cytokine expression values, mean (std. error) at rest (T0), peak effort (T1) and post-exercise (T2) in healthy male subjects, GWI and CFS. Null probability values are based on a two-tailed t test performed on Log2 transformed data, and Wilcoxon ranksum on untranformed data. **Table S4.** Cytokine expression values, mean (std. error) at rest (T0), peak effort (T1) and post-exercise (T2) in healthy female subjects, GWI and CFS. Null probability values are based on a two-tailed t test performed on Log2 transformed data, and Wilcoxon ranksum on untransformed data. Click here for file

Additional file 2: Table S5 Linear classification models separating female GWI and CFS subjects as well as male GWI and CFS subjects respectively. Performance values correspond to leave-one-out cross validation results. Click here for file

Additional file 3: Figure S1 Age dependent expression of IL-4 in healthy control subjects. In two cohorts separated by age (43 vs. 53 years), healthy control groups show significant differences in IL-4 expression while levels in CFS subjects are comparable across studies [16]. Click here for file
