# Supplementary material for: A comparison of sex-specific immune signatures in Gulf War illness and chronic fatigue syndrome
Source: BMC Immunol. 2013 Jun 25;14:29. doi: 10.1186/1471-2172-14-29 (PMC3698072; doi:10.1186/1471-2172-14-29)
Supplement: Additional file 1: Table S1 — Cohort description of mean values (std. error) with the ANOVA null probability for significance of the group effect. Table S2. Cytokine expression values, mean (std. error) at rest (T0), peak effort (T1) and post-exercise (T2) in healthy male and female subjects. Null probability values are based on a two-tailed t test performed on Log2 transformed data, and Wilcoxon ranksum on untranformed data. Table S3. Cytokine expression values, mean (std. error) at rest (T0), peak effort (T1) and post-exercise (T2) in healthy male subjects, GWI and CFS. Null probability values are based on a two-tailed t test performed on Log2 transformed data, and Wilcoxon ranksum on untranformed data. Table S4. Cytokine expression values, mean (std. error) at rest (T0), peak effort (T1) and post-exercise (T2) in healthy female subjects, GWI and CFS. Null probability values are based on a two-tailed t test performed on Log2 transformed data, and Wilcoxon ranksum on untransformed data. [file 1471-2172-14-29-S1.docx]

**Table S1**. Cohort description of mean values (std. error) with the ANOVA null probability for significance of the group effect.

| Group | Num. Subjects | Age (yrs) | BMI (kg/m^2^) | % Predicted VO2 max (ml/kg/min) |
| --- | --- | --- | --- | --- |
|  |  |  |  |  |
| Male Healthy | 21 | 40.7 (1.4) | 27.8 (0.9) | 76.0 (3.2) |
| Male GWI | 20 | 42.6 (1.4) | 28.3 (0.7) | 67.9 (2.4) |
| Male CFS | 12 | 42.1 (2.1) | 27.4 (1.2) | 63.7 (2.9) |
| *ANOVA p-value* |  | 0.27 | 0.41 | 0.04 |
|  |  |  |  |  |
| Female Healthy | 9 | 45.4 (2.1) | 31.8 (1.7) | 62.2 (9.6) |
| Female GWI | 10 | 47.2 (2.0) | 28.2 (1.4) | 56.0 (4.9) |
| Female CFS | 10 | 43.2 (2.8) | 26.9 (1.3) | 48.1 (5.7) |
| *ANOVA p-value* |  | 0.49 | 0.07 | 0.36 |

**Table S2**. Cytokine expression values, mean (std. error) at rest (T0), peak effort (T1) and post-exercise (T2) in healthy male and female subjects. Null probability values are based on a two-tailed t test performed on Log2 transformed data, and Wilcoxon ranksum on untranformed data.

|  |  |  | **t Test Log2 Transformed Data** |  |  | **Ranksum (untransformed)** |
| --- | --- | --- | --- | --- | --- | --- |
| Cytokine | Time Point | Healthy Male | Healthy Female | p Male vs Female |  | p Male vs Female |
|  |  | N=21 | N=9 |  |  |  |
| IL-1a | T0 | 10.11 (2.87) | 7.54 (2.85) | 0.84 |  | 0.72 |
|  | T1 | 12.72 (3.70) | 3.97 (1.49) | 0.18 |  | 0.08 |
|  | T2 | 11.10 (3.13) | 8.50 (1.78) | 0.28 |  | 0.55 |
|  |  |  |  |  |  |  |
| IL-1b | T0 | 21.88 (4.88) | 19.58 (4.24) | 0.80 |  | 0.89 |
|  | T1 | 27.92 (6.71) | 22.32 (7.26) | 0.60 |  | 0.68 |
|  | T2 | 22.47 (4.52) | 43.49 (25.65) | 0.90 |  | 0.82 |
|  |  |  |  |  |  |  |
| IL-2 | T0 | 12.74 (5.60) | 11.34 (5.85) | 0.41 |  | 0.79 |
|  | T1 | 17.07 (7.92) | 12.00 (6.19) | 0.43 |  | 0.89 |
|  | T2 | 8.28 (1.80) | 14.39 (8.13) | 0.51 |  | 0.93 |
|  |  |  |  |  |  |  |
| IL-4 | T0 | 14.60 (10.57) | 10.06 (5.10) | 0.66 |  | 1.00 |
|  | T1 | 30.21 (25.40) | 14.60 (5.89) | 0.23 |  | 0.43 |
|  | T2 | 19.69 (16.32) | 40.12 (29.34) | 0.18 |  | 0.40 |
|  |  |  |  |  |  |  |
| IL-5 | T0 | 13.85 (5.24) | 6.66 (3.97) | 0.89 |  | 0.21 |
|  | T1 | 18.13 (7.46) | 4.54 (1.75) | 0.08 |  | 0.17 |
|  | T2 | 11.39 (4.37) | 5.89 (3.22) | 0.89 |  | 0.16 |
|  |  |  |  |  |  |  |
| IL-6 | T0 | 9.92 (2.35) | 4.32 (0.90) | 0.24 |  | 0.50 |
|  | T1 | 10.83 (2.15) | 4.44 (0.90) | 0.08 |  | 0.25 |
|  | T2 | 9.15 (1.57) | 9.31 (2.13) | 0.84 |  | 1.00 |
|  |  |  |  |  |  |  |
| IL-8 | T0 | 13.30 (3.58) | 17.07 (10.11) | 0.44 |  | 0.30 |
|  | T1 | 17.53 (4.86) | 10.88 (5.75) | 0.33 |  | 0.44 |
|  | T2 | 17.40 (4.76) | 23.22 (11.11) | 0.97 |  | 0.82 |
|  |  |  |  |  |  |  |
| IL-10 | T0 | 14.70 (2.85) | 13.45 (1.65) | 0.50 |  | 0.50 |
|  | T1 | 19.90 (4.08) | 15.20 (1.99) | 0.99 |  | 1.00 |
|  | T2 | 14.34 (2.31) | 14.54 (1.82) | 0.30 |  | 0.50 |
|  |  |  |  |  |  |  |
| IL-12p70 | T0 | 13.99 (9.50) | 25.31 (13.11) | 0.77 |  | *0.05* |
|  | T1 | 21.73 (14.92) | 19.49 (6.25) | 0.15 |  | 0.12 |
|  | T2 | 8.15 (3.15) | 23.06 (8.95) | 0.03 |  | *0.04* |
|  |  |  |  |  |  |  |
|  |  |  |  |  |  |  |
| IL-13 | T0 | 3.44 (0.86) | 3.02 (0.61) | 0.87 |  | 0.95 |
|  | T1 | 4.48 (1.51) | 2.81 (0.46) | 0.51 |  | 0.68 |
|  | T2 | 3.17 (0.78) | 3.26 (0.75) | 0.48 |  | 0.34 |
|  |  |  |  |  |  |  |
| IL-15 | T0 | 19.30 (4.52) | 49.00 (38.60) | 0.70 |  | 0.51 |
|  | T1 | 25.89 (6.09) | 36.25 (20.92) | 0.47 |  | 0.96 |
|  | T2 | 20.01 (4.04) | 41.44 (29.51) | 0.38 |  | 0.65 |
|  |  |  |  |  |  |  |
| IL-17 | T0 | 13.50 (4.45) | 9.26 (5.22) | 0.21 |  | 0.17 |
|  | T1 | 22.93 (10.95) | 9.23 (3.59) | 0.23 |  | 0.21 |
|  | T2 | 13.12 (4.74) | 10.01 (3.39) | 0.85 |  | 0.93 |
|  |  |  |  |  |  |  |
| IL-23 | T0 | 216.59 (79.52) | 15712.14 (9946.82) | *0.00* |  | *0.00* |
|  | T1 | 272.07 (100.69) | 17280.84 (10615.43) | *0.01* |  | *0.00* |
|  | T2 | 201.21 (74.24) | 15531.71 (9875.01) | *0.01* |  | *0.00* |
|  |  |  |  |  |  |  |
| IFNg | T0 | 17.07 (6.67) | 23.29 (12.37) | 0.67 |  | 0.73 |
|  | T1 | 30.78 (12.99) | 70.87 (56.89) | 0.38 |  | 0.57 |
|  | T2 | 18.39 (7.07) | 90.41 (75.78) | 0.73 |  | 0.96 |
|  |  |  |  |  |  |  |
| TNFa | T0 | 15.21 (4.90) | 24.47 (15.46) | 0.27 |  | 0.93 |
|  | T1 | 20.85 (6.68) | 23.07 (9.87) | 0.90 |  | 1.00 |
|  | T2 | 16.95 (4.98) | 20.60 (12.05) | 0.56 |  | 1.00 |
|  |  |  |  |  |  |  |
| TNFb | T0 | 8.24 (2.69) | 3.97 (1.65) | 0.77 |  | 0.10 |
|  | T1 | 11.30 (3.60) | 8.06 (3.20) | 0.75 |  | 0.60 |
|  | T2 | 9.21 (2.21) | 7.53 (2.97) | 0.89 |  | 0.57 |

**Table S3**. Cytokine expression values, mean (std. error) at rest (T0), peak effort (T1) and post-exercise (T2) in healthy male subjects, GWI and CFS. Null probability values are based on a two-tailed t test performed on Log2 transformed data, and Wilcoxon ranksum on untranformed data.

|  |  |  |  |  | **t Test Log2 Transformed Data** | |  |  | **Ranksum (untransformed)** | | |
| --- | --- | --- | --- | --- | --- | --- | --- | --- | --- | --- | --- |
| Cytokine | Time Point | Healthy Male | GWI Male | CFS Male | p GWI vs HC | pCFS vs HC | p GWI vs CFS |  | p GWI vs HC | pCFS vs HC | pGWI vs CFS |
|  |  | N=21 | N=20 | N=12 |  |  |  |  |  |  |  |
| IL-1a | T0 | 10.11 (2.87) | 6.39 (1.60) | 9.04 (3.17) | 0.97 | 0.64 | 0.60 |  | 0.54 | 0.96 | 0.73 |
|  | T1 | 12.72 (3.70) | 22.25 (11.91) | 13.30 (3.90) | 0.66 | 0.18 | 0.33 |  | 0.91 | 0.61 | 0.52 |
|  | T2 | 11.10 (3.13) | 10.06 (4.45) | 14.17 (3.79) | 0.38 | 0.72 | 0.75 |  | 0.76 | 0.61 | 0.40 |
|  |  |  |  |  |  |  |  |  |  |  |  |
| IL-1b | T0 | 21.88 (4.88) | 19.34 (9.28) | 34.96 (13.82) | 0.09 | 0.78 | 0.14 |  | 0.07 | 0.96 | 0.24 |
|  | T1 | 27.92 (6.71) | 26.49 (10.47) | 20.44 (5.14) | 0.22 | 0.50 | 0.67 |  | 0.24 | 0.64 | 0.52 |
|  | T2 | 22.47 (4.52) | 20.40 (8.66) | 26.66 (13.21) | 0.07 | 0.37 | 0.53 |  | 0.08 | 0.14 | 0.83 |
|  |  |  |  |  |  |  |  |  |  |  |  |
| IL-2 | T0 | 12.74 (5.60) | 25.24 (11.13) | 59.40 (41.31) | 0.83 | 0.88 | 0.95 |  | 0.70 | *0.05* | 0.18 |
|  | T1 | 17.07 (7.92) | 22.10 (7.91) | 72.58 (38.59) | 0.67 | *0.03* | 0.07 |  | 0.65 | *0.02* | 0.11 |
|  | T2 | 8.28 (1.80) | 21.45 (8.79) | 54.25 (33.82) | 0.77 | *0.02* | 0.07 |  | 0.61 | *0.02* | *0.03* |
|  |  |  |  |  |  |  |  |  |  |  |  |
| IL-4 | T0 | 14.60 (10.57) | 11.79 (8.47) | 245.37 (203.07) | 0.88 | 0.50 | 0.46 |  | 0.68 | 0.93 | 0.77 |
|  | T1 | 30.21 (25.40) | 6.74 (2.45) | 338.97 (317.21) | 0.67 | 0.95 | 0.76 |  | 0.61 | 0.52 | 0.55 |
|  | T2 | 19.69 (16.32) | 3.65 (0.83) | 40.66 (26.64) | 0.65 | 0.50 | 0.63 |  | 0.69 | 0.99 | 0.83 |
|  |  |  |  |  |  |  |  |  |  |  |  |
| IL-5 | T0 | 13.85 (5.24) | 22.09 (13.29) | 7.37 (3.21) | 0.45 | 0.82 | 0.18 |  | 0.82 | 0.25 | 0.18 |
|  | T1 | 18.13 (7.46) | 22.38 (10.86) | 6.19 (2.10) | 0.78 | 0.13 | 0.08 |  | 0.78 | 0.16 | 0.08 |
|  | T2 | 11.39 (4.37) | 12.77 (4.95) | 10.18 (4.76) | 0.35 | 0.62 | 0.25 |  | 0.95 | 0.31 | 0.24 |
|  |  |  |  |  |  |  |  |  |  |  |  |
| IL-6 | T0 | 9.92 (2.35) | 12.24 (3.36) | 7.88 (2.82) | 0.80 | 0.66 | 0.99 |  | 0.37 | 0.67 | 0.12 |
|  | T1 | 10.83 (2.15) | 23.17 (10.71) | 13.22 (5.66) | 0.32 | 0.66 | 0.26 |  | 0.43 | 0.61 | 0.24 |
|  | T2 | 9.15 (1.57) | 16.58 (4.78) | 11.54 (5.02) | 0.38 | 0.51 | 0.24 |  | 0.59 | 0.34 | 0.17 |
|  |  |  |  |  |  |  |  |  |  |  |  |
| IL-8 | T0 | 13.30 (3.58) | 39.64 (12.95) | 40.97 (17.28) | *0.04* | 0.24 | 0.73 |  | 0.07 | 0.29 | 0.63 |
|  | T1 | 17.53 (4.86) | 39.44 (11.90) | 20.88 (5.89) | 0.10 | 0.60 | 0.37 |  | 0.12 | 0.61 | 0.42 |
|  | T2 | 17.40 (4.76) | 44.26 (15.23) | 36.70 (16.35) | 0.12 | 0.47 | 0.61 |  | 0.18 | 0.78 | 0.55 |
|  |  |  |  |  |  |  |  |  |  |  |  |
| IL-10 | T0 | 14.70 (2.85) | 11.63 (2.72) | 14.96 (3.33) | 0.20 | 0.88 | 0.22 |  | 0.16 | 0.69 | 0.18 |
|  | T1 | 19.90 (4.08) | 11.46 (2.03) | 20.48 (5.58) | 0.06 | 0.93 | 0.20 |  | 0.14 | 0.93 | 0.13 |
|  | T2 | 14.34 (2.31) | 11.74 (2.47) | 17.75 (2.91) | 0.25 | 0.35 | 0.07 |  | 0.27 | 0.25 | 0.07 |
|  |  |  |  |  |  |  |  |  |  |  |  |
| IL-12p70 | T0 | 13.99 (9.50) | 8.37 (3.17) | 29.71 (25.43) | 0.98 | 0.76 | 0.76 |  | 0.42 | 0.60 | 0.77 |
|  | T1 | 21.73 (14.92) | 5.80 (1.63) | 20.85 (16.19) | 0.64 | 0.54 | 0.70 |  | 0.14 | 0.29 | 0.98 |
|  | T2 | 8.15 (3.15) | 8.44 (3.79) | 31.73 (21.10) | 0.97 | 0.94 | 0.92 |  | 0.34 | 0.27 | 0.73 |
|  |  |  |  |  |  |  |  |  |  |  |  |
| IL-13 | T0 | 3.44 (0.86) | 12.76 (4.39) | 6.38 (2.12) | *0.03* | 0.30 | 0.39 |  | 0.16 | 0.60 | 0.38 |
|  | T1 | 4.48 (1.51) | 10.08 (4.03) | 7.76 (3.39) | 0.25 | 0.57 | 0.36 |  | 0.58 | 0.97 | 0.82 |
|  | T2 | 3.17 (0.78) | 12.97 (5.89) | 6.68 (2.43) | 0.06 | 0.17 | 0.72 |  | 0.14 | 0.35 | 0.70 |
|  |  |  |  |  |  |  |  |  |  |  |  |
| IL-15 | T0 | 19.30 (4.52) | 15.46 (5.06) | 100.76 (85.70) | 0.65 | 0.28 | 0.28 |  | 0.35 | 0.90 | 0.45 |
|  | T1 | 25.89 (6.09) | 21.47 (7.41) | 20.60 (6.16) | 0.29 | 0.55 | 0.73 |  | 0.19 | 0.47 | 0.77 |
|  | T2 | 20.01 (4.04) | 16.18 (5.79) | 79.12 (67.14) | 0.16 | 0.92 | 0.33 |  | 0.10 | 0.54 | 0.55 |
|  |  |  |  |  |  |  |  |  |  |  |  |
| IL-17 | T0 | 13.50 (4.45) | 17.66 (7.49) | 13.24 (4.67) | 0.71 | 0.47 | 0.56 |  | 0.87 | 0.49 | 0.63 |
|  | T1 | 22.93 (10.95) | 21.39 (11.58) | 18.93 (6.39) | 0.34 | 0.51 | 0.78 |  | 0.49 | 0.64 | 0.34 |
|  | T2 | 13.12 (4.74) | 10.52 (3.28) | 8.97 (2.50) | 0.86 | 0.64 | 0.55 |  | 0.64 | 0.81 | 0.71 |
|  |  |  |  |  |  |  |  |  |  |  |  |
| IL-23 | T0 | 216.59 (79.52) | 524.74 (184.43) | 1059.06 (657.14) | 0.06 | *0.01* | 0.29 |  | 0.07 | *0.01* | 0.34 |
|  | T1 | 272.07 (100.69) | 672.03 (257.72) | 1446.12 (827.79) | 0.14 | 0.07 | 0.51 |  | 0.11 | 0.06 | 0.55 |
|  | T2 | 201.21 (74.24) | 418.77 (152.21) | 1369.43 (731.23) | 0.07 | *0.01* | 0.19 |  | 0.10 | *0.02* | 0.21 |
|  |  |  |  |  |  |  |  |  |  |  |  |
|  |  |  |  |  |  |  |  |  |  |  |  |
| IFNg | T0 | 17.07 (6.67) | 56.21 (28.56) | 245.55 (221.19) | 0.57 | 0.82 | 0.60 |  | 0.20 | 1.00 | 0.41 |
|  | T1 | 30.78 (12.99) | 65.69 (27.16) | 55.29 (29.61) | 0.30 | 0.34 | 0.87 |  | 0.11 | 0.59 | 0.42 |
|  | T2 | 18.39 (7.07) | 64.34 (26.32) | 71.19 (50.71) | 0.58 | 0.69 | 0.49 |  | 0.22 | 0.81 | 0.36 |
|  |  |  |  |  |  |  |  |  |  |  |  |
| TNFa | T0 | 15.21 (4.90) | 20.52 (8.16) | 30.72 (14.05) | 0.19 | 0.11 | 0.50 |  | 0.94 | 0.47 | 0.50 |
|  | T1 | 20.85 (6.68) | 28.73 (13.10) | 29.72 (14.85) | 0.86 | 0.83 | 0.95 |  | 0.78 | 0.96 | 0.98 |
|  | T2 | 16.95 (4.98) | 28.11 (13.94) | 28.87 (16.81) | 0.40 | 0.24 | 0.56 |  | 0.92 | 0.51 | 0.46 |
|  |  |  |  |  |  |  |  |  |  |  |  |
| TNFb | T0 | 8.24 (2.69) | 29.00 (10.39) | 19.38 (11.37) | 0.07 | 0.22 | 0.40 |  | 0.23 | 0.69 | 0.38 |
|  | T1 | 11.30 (3.60) | 31.82 (11.75) | 27.62 (16.47) | 0.47 | 0.55 | 0.91 |  | 0.97 | 0.69 | 0.98 |
|  | T2 | 9.21 (2.21) | 34.97 (12.19) | 16.63 (8.52) | 0.12 | 0.78 | 0.29 |  | 0.72 | 0.35 | 0.44 |

**Table S4**. Cytokine expression values, mean (std. error) at rest (T0), peak effort (T1) and post-exercise (T2) in healthy female subjects, GWI and CFS. Null probability values are based on a two-tailed t test performed on Log2 transformed data, and Wilcoxon ranksum on untranformed data.

|  |  |  |  |  | **t Test Log2 Transformed Data** | |  |  | **Ranksum (untransformed)** | | |
| --- | --- | --- | --- | --- | --- | --- | --- | --- | --- | --- | --- |
| Cytokine | Time Point | Healthy Female | GWI Female | CFS Female | p GWI vs HC | pCFS vs HC | pGWI vs CFS |  | p GWI vs HC | pCFS vs HC | pGWI vs CFS |
|  |  | N=9 | N=10 | N=10 |  |  |  |  |  |  |  |
| IL-1a | T0 | 7.54 (2.85) | 4.41 (1.14) | 12.05 (3.60) | 0.96 | 0.42 | 0.35 |  | 0.70 | 0.21 | 0.08 |
|  | T1 | 3.97 (1.49) | 8.67 (3.18) | 11.87 (2.95) | 0.21 | 0.17 | 0.85 |  | 0.25 | 0.04 | 0.31 |
|  | T2 | 8.50 (1.78) | 2.99 (0.95) | 10.91 (2.94) | 0.34 | 0.91 | 0.28 |  | 0.02 | 0.67 | 0.03 |
|  |  |  |  |  |  |  |  |  |  |  |  |
| IL-1b | T0 | 19.58 (4.24) | 23.75 (9.49) | 17.23 (4.61) | 0.39 | 1.00 | 0.35 |  | 0.86 | 0.55 | 0.68 |
|  | T1 | 22.32 (7.26) | 35.57 (19.32) | 13.40 (2.49) | 0.43 | 0.70 | 0.53 |  | 0.60 | 0.78 | 1.00 |
|  | T2 | 43.49 (25.65) | 14.37 (4.40) | 12.50 (1.95) | 0.29 | 0.59 | 0.37 |  | 0.32 | 0.32 | 0.85 |
|  |  |  |  |  |  |  |  |  |  |  |  |
| IL-2 | T0 | 11.34 (5.85) | 26.09 (16.28) | 10.93 (1.62) | 0.80 | 0.24 | 0.36 |  | 0.62 | 0.12 | 0.34 |
|  | T1 | 12.00 (6.19) | 36.94 (18.38) | 9.45 (1.69) | 0.23 | 0.33 | 0.47 |  | 0.39 | 0.25 | 1.00 |
|  | T2 | 14.39 (8.13) | 32.99 (15.82) | 9.33 (1.72) | 0.68 | 0.37 | 0.72 |  | 0.37 | 0.60 | 0.55 |
|  |  |  |  |  |  |  |  |  |  |  |  |
| IL-4 | T0 | 10.06 (5.10) | 16.75 (8.78) | 1.90 (0.33) | 0.74 | 0.07 | *0.04* |  | 0.56 | 0.32 | 0.12 |
|  | T1 | 14.60 (5.89) | 31.18 (23.26) | 1.75 (0.24) | 0.93 | *0.02* | *0.02* |  | 0.72 | 0.02 | 0.02 |
|  | T2 | 40.12 (29.34) | 15.21 (4.96) | 1.93 (0.20) | 0.76 | 0.09 | *0.01* |  | 0.50 | 0.19 | 0.01 |
|  |  |  |  |  |  |  |  |  |  |  |  |
| IL-5 | T0 | 6.66 (3.97) | 10.08 (3.48) | 2.89 (0.76) | 0.09 | 0.21 | *0.01* |  | 0.02 | 0.45 | 0.01 |
|  | T1 | 4.54 (1.75) | 9.63 (2.93) | 3.21 (0.67) | *0.03* | 0.40 | *0.01* |  | 0.02 | 0.97 | 0.01 |
|  | T2 | 5.89 (3.22) | 6.02 (1.36) | 3.44 (0.86) | 0.33 | 0.44 | 0.08 |  | 0.05 | 1.00 | 0.08 |
|  |  |  |  |  |  |  |  |  |  |  |  |
| IL-6 | T0 | 4.32 (0.90) | 9.92 (4.92) | 4.86 (1.07) | 0.32 | 0.96 | 0.37 |  | 0.56 | 0.97 | 0.50 |
|  | T1 | 4.44 (0.90) | 9.47 (3.41) | 6.05 (1.83) | 0.22 | 0.75 | 0.41 |  | 0.45 | 0.90 | 0.52 |
|  | T2 | 9.31 (2.13) | 10.26 (2.82) | 5.00 (1.06) | 0.93 | 0.22 | 0.21 |  | 0.97 | 0.16 | 0.16 |
|  |  |  |  |  |  |  |  |  |  |  |  |
| IL-8 | T0 | 17.07 (10.11) | 43.99 (24.08) | 3.34 (0.81) | 0.16 | 0.49 | *0.02* |  | 0.24 | 0.66 | 0.09 |
|  | T1 | 10.88 (5.75) | 53.86 (21.34) | 3.95 (1.13) | *0.04* | 0.28 | *0.00* |  | 0.07 | 0.46 | 0.01 |
|  | T2 | 23.22 (11.11) | 50.89 (25.16) | 6.00 (2.30) | 0.17 | 0.27 | *0.01* |  | 0.21 | 0.32 | 0.01 |
|  |  |  |  |  |  |  |  |  |  |  |  |
| IL-10 | T0 | 13.45 (1.65) | 8.55 (1.86) | 11.19 (1.93) | 0.18 | 0.24 | 0.26 |  | 0.08 | 0.37 | 0.38 |
|  | T1 | 15.20 (1.99) | 9.02 (2.18) | 11.45 (1.56) | 0.16 | 0.14 | 0.25 |  | 0.03 | 0.35 | 0.31 |
|  | T2 | 14.54 (1.82) | 13.04 (2.92) | 10.86 (1.67) | 0.28 | 0.14 | 0.81 |  | 0.48 | 0.35 | 0.71 |
|  |  |  |  |  |  |  |  |  |  |  |  |
| IL-12p70 | T0 | 25.31 (13.11) | 29.99 (17.35) | 11.12 (5.51) | 0.85 | 0.39 | 0.49 |  | 0.83 | 0.25 | 0.52 |
|  | T1 | 19.49 (6.25) | 57.39 (43.66) | 13.87 (5.68) | 0.96 | 0.25 | 0.29 |  | 0.97 | 0.40 | 0.47 |
|  | T2 | 23.06 (8.95) | 9.54 (2.97) | 9.07 (4.13) | 0.11 | 0.11 | 0.71 |  | 0.22 | 0.08 | 0.85 |
|  |  |  |  |  |  |  |  |  |  |  |  |
| IL-13 | T0 | 3.02 (0.61) | 5.50 (2.56) | 3.51 (0.58) | 0.59 | 0.49 | 0.98 |  | 0.89 | 0.72 | 0.73 |
|  | T1 | 2.81 (0.46) | 10.58 (5.31) | 2.95 (0.52) | 0.36 | 0.99 | 0.37 |  | 0.95 | 0.97 | 0.57 |
|  | T2 | 3.26 (0.75) | 9.50 (5.29) | 3.43 (0.57) | 0.32 | 0.37 | 0.71 |  | 0.89 | 0.95 | 0.62 |
|  |  |  |  |  |  |  |  |  |  |  |  |
| IL-15 | T0 | 49.00 (38.60) | 87.43 (42.07) | 12.24 (1.72) | 0.68 | 0.54 | 0.96 |  | 0.29 | 0.72 | 0.21 |
|  | T1 | 36.25 (20.92) | 101.16 (59.60) | 11.87 (1.75) | 0.35 | 0.64 | 0.26 |  | 0.48 | 0.51 | 0.19 |
|  | T2 | 41.44 (29.51) | 39.01 (20.99) | 9.96 (1.40) | 0.96 | 0.56 | 0.57 |  | 0.74 | 0.50 | 0.55 |
|  |  |  |  |  |  |  |  |  |  |  |  |
| IL-17 | T0 | 9.26 (5.22) | 9.37 (3.42) | 19.76 (5.12) | 0.44 | 0.13 | 0.07 |  | 0.95 | 0.07 | 0.08 |
|  | T1 | 9.23 (3.59) | 9.74 (4.16) | 21.38 (5.46) | 0.22 | 0.10 | 0.08 |  | 0.40 | 0.11 | 0.06 |
|  | T2 | 10.01 (3.39) | 10.13 (4.14) | 19.42 (6.51) | 0.83 | 0.23 | 0.12 |  | 0.68 | 0.18 | 0.16 |
|  |  |  |  |  |  |  |  |  |  |  |  |
| IL-23 | T0 | 15712.14 (9946.82) | 1609.44 (1295.35) | 8995.00 (8093.34) | 0.09 | 0.54 | 0.16 |  | 0.05 | 0.50 | 0.14 |
|  | T1 | 17280.84 (10615.43) | 1709.29 (1360.52) | 11951.74 (11006.26) | 0.08 | 0.58 | 0.12 |  | 0.04 | 0.45 | 0.11 |
|  | T2 | 15531.71 (9875.01) | 1408.67 (1184.48) | 10909.46 (10295.22) | *0.03* | 0.40 | 0.06 |  | 0.02 | 0.36 | 0.08 |
|  |  |  |  |  |  |  |  |  |  |  |  |
| IFNg | T0 | 23.29 (12.37) | 314.76 (300.59) | 4.85 (2.45) | 0.65 | 0.17 | 0.79 |  | 0.84 | 0.24 | 0.55 |
|  | T1 | 70.87 (56.89) | 379.29 (351.58) | 4.37 (1.81) | 0.86 | 0.19 | 0.57 |  | 0.78 | 0.36 | 0.14 |
|  | T2 | 90.41 (75.78) | 168.02 (144.99) | 3.43 (1.70) | 0.49 | 0.15 | 0.99 |  | 1.00 | 0.11 | 0.31 |
|  |  |  |  |  |  |  |  |  |  |  |  |
| TNFa | T0 | 24.47 (15.46) | 54.50 (23.00) | 6.27 (1.66) | 0.23 | 0.25 | 0.07 |  | 0.21 | 0.45 | 0.05 |
|  | T1 | 23.07 (9.87) | 55.77 (25.18) | 6.66 (1.00) | 0.44 | 0.46 | 0.09 |  | 0.72 | 0.50 | 0.21 |
|  | T2 | 20.60 (12.05) | 38.88 (15.77) | 4.75 (1.37) | 0.29 | 0.17 | 0.06 |  | 0.28 | 0.32 | 0.02 |
|  |  |  |  |  |  |  |  |  |  |  |  |
| TNFb | T0 | 3.97 (1.65) | 44.45 (32.81) | 5.65 (2.09) | 0.47 | 0.79 | 0.63 |  | 0.16 | 0.56 | 0.23 |
|  | T1 | 8.06 (3.20) | 58.94 (40.71) | 7.62 (2.11) | 0.91 | 0.46 | 0.67 |  | 0.45 | 0.92 | 0.55 |
|  | T2 | 7.53 (2.97) | 36.23 (19.40) | 4.86 (1.33) | 0.18 | 0.50 | 0.14 |  | 0.24 | 0.97 | 0.24 |
