# Supplementary material for: A comparison of sex-specific immune signatures in Gulf War illness and chronic fatigue syndrome
Source: BMC Immunol. 2013 Jun 25;14:29. doi: 10.1186/1471-2172-14-29 (PMC3698072; doi:10.1186/1471-2172-14-29)
Supplement: Additional file 2: Table S5 — Linear classification models separating female GWI and CFS subjects as well as male GWI and CFS subjects respectively. Performance values correspond to leave-one-out cross validation results. [file 1471-2172-14-29-S2.docx]

**Table S5**. Linear classification models separating female GWI and CFS subjects as well as male GWI and CFS subjects respectively. Performance values correspond to leave-one-out cross validation results.

|  | Model | Stand. Coeff. | Assigned GWI | Assigned CFS | Total |  |  | Accuracy | NPV | PPV | Specificity | Sensitivity |
| --- | --- | --- | --- | --- | --- | --- | --- | --- | --- | --- | --- | --- |
| *Male Subjects* |  |  |  |  |  |  |  |  |  |  |  |  |
| GWI vs CFS males at T0-T2 | *No model identified* | |  |  |  | True GWI male |  | NA | NA | NA | NA | NA |
|  |  |  |  |  |  | True CFS male |  |  |  |  |  |  |
| *** All possible subsets* |  |  |  |  |  |  |  |  |  |  |  |  |
| GWI vs CFS males at T0-T2 | TNFb T0 | 0.41 | 13 | 7 | 20 | True GWI male |  | 0.66 | 0.76 | 0.53 | 0.65 | 0.67 |
|  | IL-1b T0 | -0.65 | 4 | 8 | 12 | True CFS male |  |  |  |  |  |  |
|  |  |  |  |  |  |  |  |  |  |  |  |  |
| GWI vs CFS males at T0-T2 | TNFb_T0 | 1.13 | 19 | 1 | 20 | True GWI male |  | 0.94 | 0.95 | 0.92 | 0.95 | 0.92 |
|  | IL-1b_T0 | -1.31 | 1 | 11 | 12 | True CFS male |  |  |  |  |  |  |
|  | IL-2_T1 | -1.97 |  |  |  |  |  |  |  |  |  |  |
|  | IL-6_T1 | 1.92 |  |  |  |  |  |  |  |  |  |  |
|  |  |  |  |  |  |  |  |  |  |  |  |  |
| *Female Subjects* |  |  |  |  |  |  |  |  |  |  |  |  |
| GWI vs CFS females at T0 | Il-1b T0 | -0.83 | 9 | 1 | 10 | True GWI female | | 0.90 | 0.90 | 0.90 | 0.90 | 0.90 |
|  | Il-5 T0 | 1.15 | 1 | 9 | 10 | True CFS female | |  |  |  |  |  |
|  |  |  |  |  |  |  |  |  |  |  |  |  |
| GWI vs CFS females at T1 | IL-1a T1 | 1.10 | 10 | 0 | 10 | True GWI female | | 1.00 | 1.00 | 1.00 | 1.00 | 1.00 |
|  | Il-5 T1 | 0.89 | 0 | 10 | 10 | True CFS female | |  |  |  |  |  |
|  | Il-8 T1 | 0.65 |  |  |  |  |  |  |  |  |  |  |
|  | Il-15 T1 | 1.01 |  |  |  |  |  |  |  |  |  |  |
|  | Il-17 T1 | -2.53 |  |  |  |  |  |  |  |  |  |  |
|  |  |  |  |  |  |  |  |  |  |  |  |  |
|  |  |  |  |  |  |  |  |  |  |  |  |  |
|  |  |  |  |  |  |  |  |  |  |  |  |  |
| GWI vs CFS females at T2 | Il-1b T2 | 0.68 | 10 | 0 | 10 | True GWI female | | 1.00 | 1.00 | 1.00 | 1.00 | 1.00 |
|  | Il-2 T2 | -2.00 | 0 | 10 | 10 | True CFS female | |  |  |  |  |  |
|  | Il-15 T2 | -1.37 |  |  |  |  |  |  |  |  |  |  |
|  | Il-17 T2 | 1.88 |  |  |  |  |  |  |  |  |  |  |
|  | Il-23 T2 | 1.66 |  |  |  |  |  |  |  |  |  |  |
|  |  |  |  |  |  |  |  |  |  |  |  |  |
| GWI vs CFS females at T0-T2 | Il-10 T0 | 12.81 | 10 | 0 | 10 | True CFS female | | 1.00 | 1.00 | 1.00 | 1.00 | 1.00 |
|  | Il-12p70 T0 | -2.86 | 0 | 10 | 10 | True GWI female | |  |  |  |  |  |
|  | IL-1a T1 | 12.48 |  |  |  |  |  |  |  |  |  |  |
|  | Il-8 T1 | -12.86 |  |  |  |  |  |  |  |  |  |  |
|  | Il-15 T1 | -12.23 |  |  |  |  |  |  |  |  |  |  |
|  | Il-17 T1 | 15.04 |  |  |  |  |  |  |  |  |  |  |
|  | TNFb T1 | -24.22 |  |  |  |  |  |  |  |  |  |  |
|  | IL-1a T2 | -2.50 |  |  |  |  |  |  |  |  |  |  |
|  | Il-6 T2 | 1.45 |  |  |  |  |  |  |  |  |  |  |
|  | Il-13 T2 | 10.77 |  |  |  |  |  |  |  |  |  |  |
|  | Il-17 T2 | 12.95 |  |  |  |  |  |  |  |  |  |  |
|  |  |  |  |  |  |  |  |  |  |  |  |  |
