# Supplementary figures and images for: A comparison of sex-specific immune signatures in Gulf War illness and chronic fatigue syndrome
Source: BMC Immunol. 2013 Jun 25;14:29. doi: 10.1186/1471-2172-14-29 (PMC3698072; doi:10.1186/1471-2172-14-29)

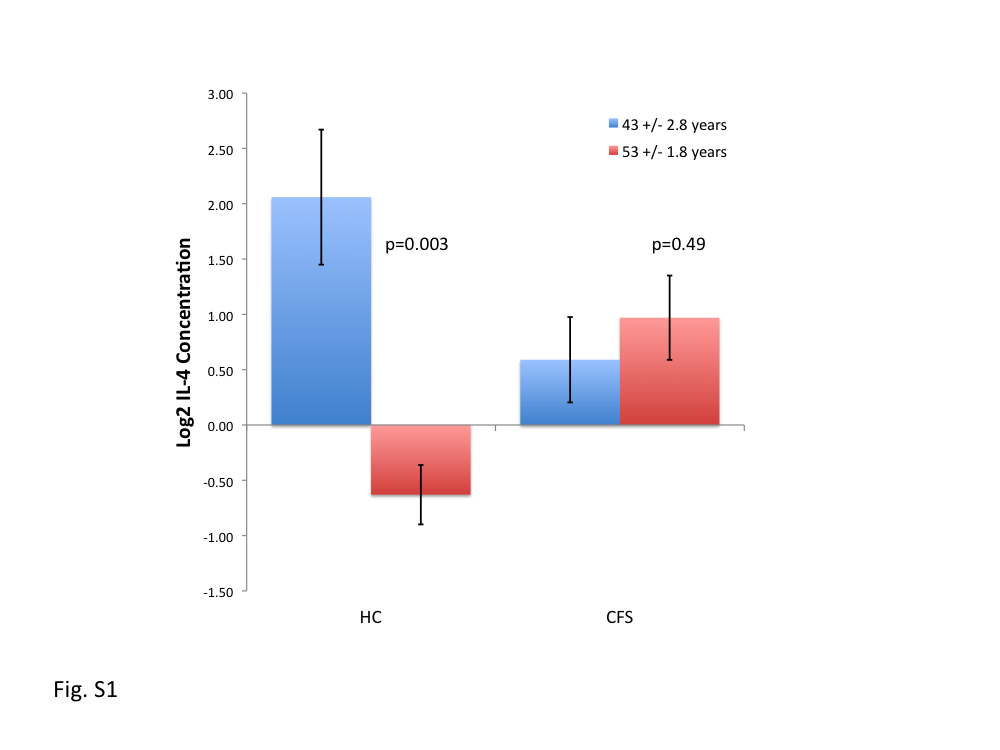

Supplement: Additional file 3: Figure S1 — Age dependent expression of IL-4 in healthy control subjects. In two cohorts separated by age (43 vs. 53 years), healthy control groups show significant differences in IL-4 expression while levels in CFS subjects are comparable across studies [16]. [file 1471-2172-14-29-S3.tiff]
